# Enduring benefits of exercise after spinal cord injury: Insights from a case study

**DOI:** 10.4102/ajod.v14i0.1633

**Published:** 2025-06-25

**Authors:** Candace Vermaak, Lovemore Kunorozva

**Affiliations:** 1 Department of Exercise, Sport and Lifestyle Medicine, Faculty of Medicine and Health Sciences, Stellenbosch University, Cape Town, South Africa; 2 Brigham and Women’s Hospital, Division of Sleep and Circadian Disorders, Faculty of Medicine, Harvard University, Boston, United States of America; 3 Broad Institute, Molecular and Population Genetics Program, Faculty of Medicine, Massachusetts Institute of Technology, Cambridge, United States of America; 4 Center for Genomic Medicine, Faculty of Medicine, Harvard University, Boston, United States of America

**Keywords:** spinal cord injury, physical activity, sport: rehabilitation, exercise

## Abstract

**Introduction:**

Spinal cord injuries (SCIs), while relatively rare, profoundly alter the lives of those affected. Among the diverse causes of SCI, traumatic sporting injuries represent 8.7% of newly reported cases. Despite the life-altering consequences of SCI, physical activity (PA) can mitigate some of these impacts.

**Patient presentation:**

This case study highlights the long-term benefits of sustained PA and its crucial role in fostering a cascade of positive outcomes post-SCI. We present the case of an elite South African athlete, ‘Jim’, who sustained a complete SCI at the C6 level at the age of 16 in 2012 following a fall during dismount in a gymnastics competition. Jim’s rehabilitation journey began shortly after the injury, encompassing formal inpatient care, alternative therapies and ultimately a return to competitive sports. By 2015, Jim resumed athletic activities, beginning with shot put and discus before transitioning to wheelchair racing, which became a pivotal aspect of his recovery.

**Management and outcome:**

The case emphasises how sustained PA led to Jim’s significant physical and mental improvements, such as weight loss, enhanced self-esteem, increased mobility and greater independence in daily living. Moreover, sport provided Jim with a renewed sense of purpose and direction.

**Conclusion:**

This case underscores the importance of creating ongoing opportunities for individuals with SCI to continue their recovery long after discharge from inpatient rehabilitation.

**Contribution:**

This case study illustrates how sustained PA contributed to a cascade of positive outcomes in Jim’s recovery following SCI, offering valuable insights from his lived experience.

## Introduction

Spinal cord injuries (SCIs), though infrequent, account for a significant burden on those affected. Globally, motor vehicle accidents are the leading cause of SCIs, accounting for approximately 38% of cases (Bennett, Das & Emmady 2024), while sports-related injuries contribute to an estimated 8.7% – 9% of new cases (Bennett et al. 2024; Boden & Jarvis 2008). In contrast, within developing countries such as South Africa, assault is the most common cause responsible for up to 60% of SCIs, followed by transport-related injuries (26%) and falls (12%) (Joseph et al. 2017). The severity of SCI largely depends on the injury’s location and completeness (Vincent et al. 2014), with cervical injuries being among the most severe because of their potential to impair motor and sensory function in both the upper and lower limbs, the trunk and respiratory system (Szeliga et al. 2022). A SCI often results in permanent damage, interrupting communication between the brain and body, leading to a wide range of symptoms from loss of sensation and muscular control to impaired autonomic function (Chiou et al. 2022). These impairments significantly reduce independence, health and quality of life (QoL) and increase the risk of secondary health conditions (SHCs), contributing to early mortality (Conti et al. 2020).

Despite the profound impact of SCI, physical activity (PA) offers an avenue to mitigate these effects (Vermaak et al. 2022). Physical activity has been shown to enhance emotional well-being, improve functional status, including coordination, muscle strength and endurance, and facilitate the completion of activities of daily living (Szeliga et al. 2022). Furthermore, PA helps prevent chronic health conditions (CHCs) such as heart disease, stroke and type 2 diabetes, which are often exacerbated by a sedentary lifestyle (Soriano et al. 2022). While much research has focused on the short-term benefits of sport and PA (Baehr et al. 2022, Rauch et al. 2013, Williams et al. 2014), this case study illustrates the enduring advantages of long-term PA and its essential role in ensuring ongoing recovery post-SCI.

### Study design

This case study employed a qualitative, descriptive methodology to explore the long-term benefits of sustained PA in the rehabilitation and recovery of an individual with a SCI. The case study design was chosen to provide an in-depth understanding of the PA experiences and outcomes of a single participant, offering rich, contextual insights that quantitative methods cannot capture (Yin 2018).

### Participant selection

The participant, referred to as ‘Jim’ for confidentiality, was purposively selected because of his unique experience as an elite athlete who sustained a complete SCI following a fall during a gymnastics dismount. The case was selected as Jim demonstrated sustained engagement in PA throughout his rehabilitation journey. Jim was an existing acquaintance of the lead researcher, identified through shared involvement in local adaptive sports networks, which facilitated initial contact and recruitment. This selection aligns with the intrinsic case study approach (Creswell & Poth 2018), where the subject is chosen for the unique and illustrative value of their story, particularly Jim’s remarkable resilience and sustained engagement in rehabilitation and sport. His experience provides insight into long-term SCI recovery within a low-resource context, making it a valuable lens for exploring rehabilitation beyond formal care environments.

### Data collection

Data were collected through a single in-depth, semi-structured interview with Jim. The interview was conducted via video call on Zoom, and audio-recorded with Jim’s consent. The interview focused on detailed narratives about his lived experiences, challenges and progress throughout his rehabilitation journey. Open-ended questions were used to explore his perspectives on PA, mental health, chronic conditions, SHCs and QoL post-SCI.

### Data analysis

Inductive thematic analysis was used to analyse the qualitative data (Braun & Clarke 2006). The analysis followed these steps:

- **Familiarisation**: Interview recordings, notes and transcripts were reviewed multiple times to gain an overall understanding of the data.
- **Coding**: Relevant segments of the data were systematically coded to identify patterns and recurring themes related to PA and recovery outcomes.
- **Theme development**: Codes were grouped into broader themes, such as ‘physical health improvements’, ‘psychosocial benefits of sport’ and ‘barriers to sustained activity’.
- **Narrative synthesis**: A detailed case narrative was constructed to illustrate the key findings and their implications for rehabilitation.

## Ethical considerations

Ethical approval was obtained from the Stellenbosch University Human Research Ethics Committee review board (HREC Number: HEA-2023-27120). Written informed consent was obtained from Jim, and measures were taken to ensure confidentiality through the use of a pseudonym.

### Trustworthiness and rigour

The credibility of the findings was enhanced through triangulation, comparing interview data with field notes to validate key themes, as well as through study member checking, where Jim reviewed and confirmed the accuracy of the interpreted findings. In addition, peer debriefing with colleagues provided external insights into the analysis and helped minimise potential researcher bias, which may be linked to the lead researcher’s prior clinical experience in rehabilitation settings and personal interest in PA post-SCI (Lincoln & Guba 1985; Shenton 2004). Peer debriefing involved colleagues with expertise in qualitative research and rehabilitation science independent of the study who reviewed selected data and themes, offering critical feedback to improve clarity, reduce potential bias and ensure the findings accurately reflected the participant’s lived experience.

### Transferability

Transferability was supported by providing a thick description of Jim’s background, injury and rehabilitation context. While this is a single case, such rich contextualisation enables readers to assess the relevance of the findings to other similar contexts (Lincoln & Guba 1985).

### Dependability

Dependability was addressed by maintaining an audit trail of all decisions made during the research process, including coding frameworks, inductive thematic development and analytic memos. This allows others to follow the progression of analysis and ensures the process is traceable and consistent (Nowell et al. 2017).

### Confirmability

Confirmability was strengthened by documenting reflexive notes and engaging in regular reflection to acknowledge and manage personal biases. Peer debriefing also contributed to ensuring that the findings were shaped by the participant’s data rather than researcher assumptions (Lincoln & Guba 1985).

### Relevance to rehabilitation

The methodology outlines the procedures undertaken to explore the role of sustained PA in SCI recovery. By combining personal narratives such as the neurological level of injury, SHCs and mobility status with clinical data, this case study offers valuable insights into how ongoing PA influences physical, psychological and social outcomes post-SCI. These findings have implications for designing personalised rehabilitation programmes that extend beyond inpatient care to support long-term recovery and QoL.

## Patient presentation

Jim, an elite South African gymnast, sustained a complete SCI C6 at the age of 16 during a Junior Olympic training competition in 2012. The injury occurred when he lost focus on the parallel bars, resulting in a headfirst fall that left him conscious but in immense pain. Despite his desire to complete his routine, Jim instinctively remained still, aware of the severity of his injury.

Before the injury, Jim had been experiencing burnout and was coping with the recent loss of his godfather, factors that may have contributed to his lack of preparation for the competition. With an 8-year career in gymnastics, including provincial and national-level competition, Jim’s life was deeply intertwined with his sport.

## Management and outcome

### Jim’s rehabilitation journey: Interventions, challenges and progress

Immediately post Jim’s injury, he spent a week in intensive care before beginning his rehabilitation journey. During this initial phase, he struggled to come to terms with his condition, isolating himself from others with disabilities and resisting acceptance of his new reality. He feared that acknowledging his injury meant surrendering to it, holding onto the hope that he might walk again. Medical professionals repeatedly advised him to accept his circumstances, but this proved to be a significant emotional challenge. Beyond the psychological burden, Jim found the loss of independence particularly difficult. Simple tasks, such as relying on others for feeding, adhering to a strict bathroom routine and the fear of falling because of loss of balance, made him feel as though his autonomy and dignity had been unfairly taken from him. However, his optimism and background in gymnastics provided a foundation for resilience. His physical fitness prior to the injury contributed to his ability to persevere through these early challenges.

Jim was initially treated at a leading rehabilitation centre in Cape Town, where his medical aid allowed him access to high-quality care. His rehabilitation team prioritised early intervention to capitalise on his residual muscle strength. Although initially placed in an electric wheelchair, Jim’s determination led him to transition to a manual wheelchair within 6 weeks, driven by his desire to regain independence despite facing his mental challenges and having to accept that he might not walk again. His optimism, coupled with his background in gymnastics, helped him persevere during this challenging time, as he remained physically fit and strong despite the SCI.

Pushing his wheelchair was tough, but he knew that if he did, he would only get stronger. He tried to do everything independently, and the more he failed at the task (e.g. he would drop cutlery when eating), the more determined he was to try again. Jim would rather make his life a little bit more difficult to get stronger than take the easy way out. It is just his nature. He spent a total of 10 weeks at the rehabilitation centre in Cape Town. During this time, Jim regained some function in his triceps and wrist flexion. After 10 weeks of rehabilitation, Jim’s medical and support team concluded that further improvement in his recovery would be limited. At this critical juncture, an opportunity arose for Jim to continue his rehabilitation at the Shepherd Rehabilitation Centre in Birmingham, Alabama, US. This opportunity marked a significant turning point in Jim’s recovery, as the centre offered a fresh perspective on rehabilitation modalities and introduced him to new therapies and strategies. During his time at the Shepherd Rehabilitation Centre, Jim experienced the most substantial progress in his recovery since his SCI, including marked improvements in upper limb function, trunk control and independence in daily activities.

Although Jim experienced great gains upon returning to his residence, he found the transition challenging, as it brought forth numerous questions about his future and career. Additionally, he noticed significant weight gain, which he attributed to the routine dosage of colostrum supplements intended to aid in cellular regeneration.

In addition to his rehabilitation, between 2012 and 2014, Jim committed to several intensive therapies, dedicating 2–3 h a day, Monday to Friday, and occasionally on Saturday mornings. His regimen involved working with a diverse team, including his former gymnastics coach, a personal trainer, a bodybuilder, occupational therapists, physiotherapists and practitioners of alternative therapies. Jim pursued several alternative therapies in his recovery journey. He reported that acupuncture improved his hand function from being unable to grasp objects to regaining partial grip strength and tactile sensation. He also explored homoeopathy, colostrum supplementation (which unfortunately led to unwanted weight gain) and stem cell therapy in his effort to enhance function and overall well-being. Jim embraced every available opportunity to enhance his recovery and move closer to regaining some independence. In 2015, Jim initially participated in adapted para-athletic activities, specifically shot put and discus before later discovering and transitioning to wheelchair racing. This shift to cardiovascular-focused sports was transformative, leading to dramatic improvements in his physical health, including weight loss, enhanced metabolism, better temperature regulation and improved bowel and bladder function. These physical changes, in turn, bolstered Jim’s mental well-being, self-esteem and independence, enabling him to navigate life with greater ease and confidence.

As Jim lost weight, his wheelchair size decreased, allowing him to fit into smaller spaces and move around more easily. The lighter wheelchair also made it easier for him to load it into his car independently. Weight loss further benefitted him by reducing the strain on his shoulder joints during pressure relief exercises. Because of the level of his SCI, Jim was susceptible to both CHCs and SHCs, which, if not properly managed, could have significantly impacted his QoL. However, regular PA played a crucial role in enhancing his QoL, improving both his physical capabilities and confidence. With an improved metabolism, Jim could enjoy a diverse range of foods to support his training and recovery without the fear of gaining weight. Sport provided Jim with a sense of purpose and a goal to strive for, shifting his focus away from what he could not do, like wiggling his toes, to what he could achieve. He became dedicated to improving his performance, working on becoming faster and stronger while also recognising the importance of recovery, proper seating and the right exercises to stay injury-free. Remarkably, since incorporating PA into his daily routine, Jim did not experience any SCI-related illnesses throughout his post-injury athletic career.

## Discussion

This case study chronicles the rehabilitation and recovery journey of an athlete who sustained a complete SCI during a competitive sporting event. While sport was the initial cause of his injury, it also played a pivotal role in his long-term recovery. Although sport can serve as a catalyst for recovery, previous research indicates that younger age at the time of injury is associated with improved muscle strength and functional outcomes, likely because of greater physiological plasticity and motivation to engage in rehabilitation efforts (Thomas & Grumbles 2014). In Jim’s case, he sustained the SCI at the age of 16, when he was in peak physical condition. His medical team leveraged his fitness level to enhance his recovery process. The case highlights how early intervention, coupled with sustained PA, can lead to significant improvements in both physical and mental health outcomes post-SCI. The case also emphasises the need for ongoing opportunities for individuals with SCI to continue their recovery outside of formal rehabilitation settings while also recognising the critical role that personal resilience, determination and intrinsic motivation exemplified by Jim play in sustaining PA and achieving meaningful recovery.

Although early intervention was implemented in this case study, treating a complete SCI remains a significant challenge, often leading to limited recovery, particularly after inpatient discharge (Piazza & Schuster 2017). It also underscores the significance of patient determination and dedication in achieving better health outcomes. Jim’s journey highlights the importance of continued rehabilitation beyond the inpatient phase. As Jim transitioned from power-based sports to wheelchair racing, he not only experienced physical improvements but also a renewed sense of purpose and direction. His story illustrates how determination, resilience and sustained PA can drive recovery, even in the face of a complete SCI. This is consistent with findings that highlight the critical role of patient motivation in enhancing functional capacity during rehabilitation (Krysa et al. 2022). Additionally, evidence suggests that individuals who demonstrate high resilience at the onset of rehabilitation tend to achieve better recovery outcomes (Monden et al. 2014).

Secondary health conditions, such as respiratory infections, bladder infections and cardiovascular complications, are commonly reported in individuals with SCI, particularly those with cervical injuries (Sezer, Akkuş & Uğurlu 2015). In this case study, Jim noted that following his SCI but prior to 2015 when he began rehabilitation at the Shepherd Rehabilitation Center, he experienced one intestinal bacterial infection and bronchitis three times. He attributes the subsequent improvement in his immune function to weight loss, improved nutrition and diet, and his current level of sport and PA. Endurance sports have been shown to positively impact the management of CHCs such as obesity, diabetes, coronary heart disease and early mortality (Dhule et al. 2022; Ginis, Jörgensen & Stapleton 2012). Sports participation provided Jim with a sense of belonging, social interaction and emotional support, all of which were crucial to his recovery. Competitive sports for individuals with disabilities have expanded significantly over the past few decades, offering opportunities from developmental levels to elite competition, and have the power to transform lives (Blaauwet & Willick 2012; Ramsden et al. 2023). In this case study, Jim experienced these transformative benefits, with weight loss being one of the most impactful. This weight loss led to a series of positive outcomes, including a reduction in his wheelchair size by two sizes, which greatly improved his confidence and QoL. This aligns with research showing that cardiovascular exercise, such as hand cycling (Chiou et al. 2022) and arm-crank exercise (Farkas et al. 2021; Nightingale et al. 2018), can increase lean body mass (Vivodtzev & Taylor 2021). Additionally, improved metabolic regulation from cardiovascular exercise (Nightingale et al. 2017) allowed Jim to maintain a diverse diet to support his training and recovery without gaining weight.

Finally, while this case highlights the potential benefits of sustained PA in SCI rehabilitation, it also underscores the socio-economic disparities in access to such opportunities. Jim’s access to private healthcare, overseas interventions and a multidisciplinary support team may not be representative of the broader SCI population in South Africa, particularly those reliant on under-resourced public health services. This disparity raises important considerations for equity in rehabilitation programming and the need for scalable, community-based interventions that can be adapted to lower resource settings.

It underscores the importance of early and sustained PA, community-based support and creative use of locally available resources to promote functional recovery and long-term engagement in PA even in the absence of international facilities or elite sports programmes. Jim’s experience demonstrates that exercise, proper nutrition and SHC management can significantly enhance recovery outcomes, even in challenging circumstances. For example, sport provided Jim with a sense of purpose and a clear goal to work towards. He focused on improving his athletic performance, which included maintaining proper nutrition and recovery practices, crucial elements in preventing injuries that could lead to bed rest and rehospitalisation, potentially undoing the functional gains he had achieved. This underscores the importance of education on self-monitoring and injury prevention, especially as individuals with SCI transition from inpatient rehabilitation to managing their condition in their home environment. The education received during rehabilitation plays a vital role in shaping the health outcomes of individuals with SCI (Conti et al. 2020). However, after discharge, patients often face a critical period of adjustment, navigating the challenges of living with their disability without the constant support of a rehabilitation facility. Many are discharged before fully mastering the self-management skills necessary for independent living (Conti et al. 2020). While Jim demonstrated notable psychological resilience potentially influenced by his elite sporting background, he did not report engaging with formal mental health services during his recovery. This highlights a key consideration: not all individuals will possess the same internal coping resources, and structured mental health support may be critical for others navigating similar life-altering injuries. Therefore, in addition to community-based PA programmes, adaptive sports and peer support groups, accessible and ongoing mental health services should be considered essential components of long-term rehabilitation strategies. improvement.

## Conclusion

Twelve years post-injury, Jim continues to experience incremental improvements, highlighting the ongoing potential for recovery following a complete SCI. While treating a complete SCI remains a significant challenge, this case study demonstrates the critical role of sustained PA, coupled with a positive mindset, in promoting recovery and improving QoL. The findings underscore the need for increased access to rehabilitation opportunities and education on the benefits of PA for individuals with SCI, particularly in underserved communities. Therefore, utilising accessible and affordable approaches such as exercise, along with education on proper nutrition and SHC management, may significantly enhance recovery for many patients following SCI.
